# Salicylic Acid Enhances Growth, Photosynthetic Performance and Antioxidant Defense Activity Under Salt Stress in Two Mungbean [Vigna radiata (L.) R. Wilczek] Variety

**DOI:** 10.1080/15592324.2023.2217605

**Published:** 2023-06-08

**Authors:** Esther Ogunsiji, Caroline Umebese, Edith Stabentheiner, Emmanuel Iwuala, Victor Odjegba, Ayoola Oluwajobi

**Affiliations:** a Institute of Plant Science, University of Graz, Graz, Austria; b Department of Botany, University of Lagos, Lagos, Nigeria; c Department of Plant Science and Biotechnology, Federal University Oye Ekiti, Ekiti State, Nigeria

**Keywords:** Salinity, salicylic acid, mungbean, tolerance, enzymes, yield

## Abstract

Salt is regarded as a main cause for reduced yield under challenging conditions. Mungbean, a valuable protein crop, is sensitive to salt stress, leading to yield shortage. The growth hormone, salicylic acid (SA), enhances several processes necessary to confer salt tolerance and relieves poor agricultural yield. Seeds of mungbean were initially pretreated with SA (0.5 mM) for 4 h before sowing, while under a cumulative combination of SA + salt regimes: control, SA, 100 mM, SA +100 mM, 200 mM and SA +200 mM. Our study examined photosynthesis parameters such as photosynthetic pigment concentration, chlorophyll *a* fluorescence, protein, proline, and antioxidant enzymes in plants subjected to single and combined SA + salt stress concentrations. The result showed a greater decline in SPAD and photosynthetic quantum yield under 200 mM NaCl at 43% in Var. 145 than in Var. 155 at 32% compared to 11% in SA +100 mM and 34% in SA + 200 mM treatments in both varieties. Var. 145 was found to be more sensitive to 100 and 200 mM NaCl salt stress. In Var. 155, chlorophyll *a* and chlorophyll *b* concentrations were higher under control 52%, SA + 100 mM 49%, and SA +200 mM 42% than in Var. 145 at 51%, 38%, and 31%. Protein and proline revealed a higher content in Var. 155 in contrast to the lower activity in Var. 145. The enhanced performance of the Var. 155 exposed to SA + salt stress was followed by an increase in the activities of peroxidase (POD), CAT while the activity of MDA revealed a significant increase in Var. 145 under 100 mM 43% and 200 mM 48% NaCl treatment compared to Var. 155, which had 38% and 34%. The above results suggest that SA-treated Var. 155 confers tolerance to salt stress and is accompanied with a high osmoprotectant responses as provided by SA in Var. 155 than Var. 145. The potency of SA in providing salt tolerance capacity to plants is a future research interest to maintain sustainable yield in mungbean seedlings.

## Introduction

The increase in human population has caused a serious threat to agriculture^1^. Also, abiotic stress conditions are major factors that decrease agricultural productivity by over 50%. Salinity stress is regarded as an abiotic stress condition that hinders plant growth and development^2^. Salinization is said to be the process of increasing the concentration of the amount of total dissolved salts in water and soil, which can occur in a natural state or from anthropogenic source^1^. However, as a result of global warming, the salinized region is gradually increasing on a daily or frequent basis^2,3^. Ref. ^4^ reported that about 20% of irrigated land is adversely disrupted by salinity, and it is a key effect of the decline in crop growth in the savanna and dry regions. Salt stress has a direct negative effect on plant establishment, development, and yield as a result of its impacts on the plant morphology, biochemistry and physiology. Its effects extend above all groups of development, from seed germination to full maturity^1^. Several mechanisms underlying the effects of salinity in plants are (i) salinity-mediated hinderance in plant growth through water stress; (ii) excessive uptake of ions like Na^+^ and Cl^_^ as a result of toxicity; (iii) impaired metabolism and nutritional imbalance of mineral elements; and (iv) physiological imbalance between oxidants and antioxidants due to oxidative stress^5–9^. There is an urgent need to develop salinity tolerance crops, which is necessary to produce world food availability for the ever-growing human population. Several losses in yield of 15–90% are reported in some agricultural crops as a result of salinity stress. For example, cotton, wheat, and maize showed 28%, 15%, and 55% yield losses under mild soil saline stress, also 82% and 47% losses were recorded in tef and cotton exposed to severe salt stress^10–13^. The different sensitivity of plants to saline condition depends on their overall genetic component and their entire environment. However, screening of many varieties is vital to choose the most performed varieties with high tolerance to salt stress. Mungbean (*Vigna radiata*) is known to be a leguminous crop and a valuable source of food and can be processed into several usage. Although mungbean is not a salt-tolerant crop and only little reports have been reported on the impact of salt stress on crop development^14^. The morphological and physiological mechanisms of mungbean genotypes and salt stress have been obtained in greenhouse^15^. Salicylic acid (SA) is a vital growth hormone with a significant agronomic advantage to enhance the salt tolerance of crops^16^. SA has the capacity to modulate plant stress responses to abiotic conditions by controlling plant growth, flowering, ripening, and regulatory functions^17–19^. SA is an endogenous phenolic compound that serves as a signal sensor to regulate plant response. It preserves plant cells from the disruption of ion accumulation and cell death by managing processes like nitrogen metabolism, antioxidant defense, water stress, and photosynthesis^20^. SA plays a vital function in plant defense responses to environmental stress situations^21^ and possesses defense signaling in both saline and biotic stress^22,23^, but the specific function of SA in mitigating plant salt stress still remains limited. This study will provide a potent information on the interaction between SA and salt-tolerant ability in mungbean plants via physiological and biochemical analyses. However, an interplay of varying salt resistant/adaptive responses generated by SA in mungbean seedlings is proposed and the potential mechanism of SA-mediated plant tolerance to salt stresses. Our belief is that the hormone SA will enhance the photosynthetic efficiency of mungbean by hastening cellular processes for any stress adaptation to stabilize plant development exposed to salt stress environment.

## Materials and methods

### Plant growth conditions and screening for salinity stress tolerance

**Figure 1.**
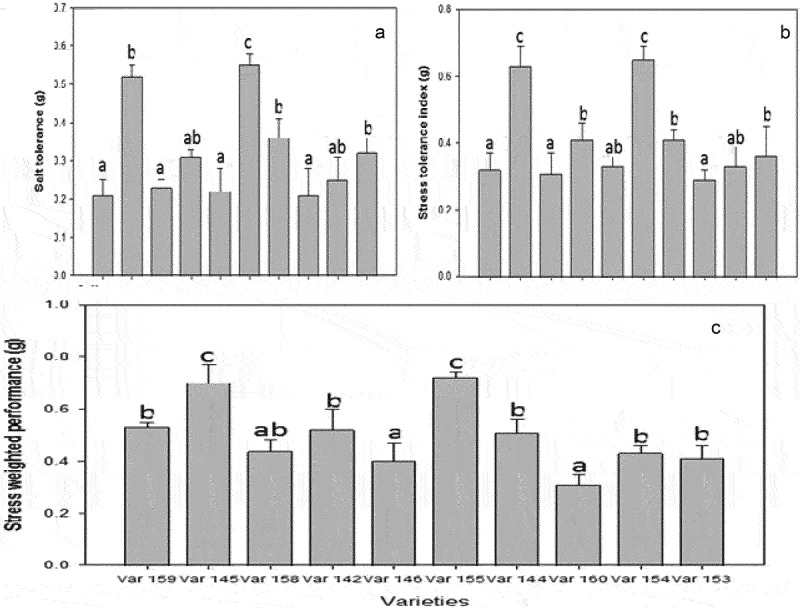
Screening of the 10 varieties of mungbean on the basis of stress indices, (a) stress tolerance, (b) stress tolerance index, and (c) stress weighed performance calculated by using relative growth stage data after 14 d of treatment with 50 mM NaCl. Different alphabets denote significant difference. Values represent means ± SE (*n* = 5).

This study is aimed at screening 10 mungbean landraces for salt stress tolerance at 50 mM NaCI concentration under in vitro conditions. All 10 landraces of mungbean were sourced from the National Horticultural Research Institute, (NIHORT), Ibadan, Nigeria, as shown in Figure 1. We believe that our work will be the first report on evaluating these mungbean landraces for salt stress resistance. The screening of different mungbean landraces for salinity was conducted at the juvenile stage using the salt-dependent nutrient solution following the procedure of ref.^24^. Salt treatment and non-salinized experimental setups were carefully maintained in the growth chamber at 26 ± 4°C at 70 ± 6% relative humidity (RH). Fourteen-day-old plantlets of mungbean were exposed to 50 mM NaCl for 12 d in a NaCl supplemented Hoagland’s medium. The media were changed every 3 dto maintain pH of the medium and a stable salt concentration. Control (0 mM NaCl) plants were maintained under the same growth condition. After 14 d of salinity treatment, mungbean seedlings were harvested and measured to estimate the relative growth rate (RGR) according to the protocol of ref.^25^ (Figure 1).

Considering the phenotypic analysis in this study, different stress indices were calculated for the 10 varieties, according to this formula: Stress Tolerance Index (STI) = STI = YC/Yav × YS/Yav^26^. Salt Tolerance (ST) = S/C = YS/YC^27^. Stress weighed performance (SWP) = YS/√YC^28^ where Yc represents the biomass of a cultivar exposed to non-saline conditions, Ys stands for weight of a cultivar exposed to saline conditions while Yav denotes relative average weight for all the cultivars exposed to saline/nonsaline conditions.

### SA application and salt treatment

A further experiment was performed in the greenhouse facility at the Institute of Biology, University of Graz, Austria (26° 55′ N latitude, 80° 59′ E longitude and at an altitude of 113 m in subtropical climate). Seeds of two (Var. 145 and Var. 155) varieties that performed best from the screened 10 varieties under 50 mM NaCI concentration were selected for the entire experiment. Application of SA was conducted through the method of seed priming by immersing the seeds of Var. 145 and Var. 155 in SA solution of 0.5 mM for 4 h before sowing while the seeds to be considered as control were only kept in water. We selected the concentration of SA on the basis of maximum seed germination rate when exposed to SA pretreatment. Afterward, the seeds were surface sterilized and sown on a filter paper moist with double distilled water, then kept in Petri dishes at 26 ± 2°C for 1 week. The seedlings were now transferred to plastic bowl of 12 cm height for further growth and development. Salt-treated and non-salt-treated setups were constantly checked in the growth chamber at 28 ± 3°C at 70 ± 4% relative humidity (RH). Water was applied regularly until seedling emergence. Seven-day plants of mungbean were exposed to different salt concentration levels: 100 and 200 mM NaCl for 3 weeks by growing the plantlets in NaCl medium. The supplemented medium was always changed every 3 dinterval in order to stabilize the pH of the medium and salt concentration. We selected 200 mM NaCl salt concentration for time point in this experiment because the mungbean seedlings showed a noticeable stress phenotypic changes. The combinations of SA and concentrations of salt applied to the soil culminated in a total of six treatments employed to elucidate the synergistic effects of SA and salt on the growth pattern and physiology of the mungbean seedlings. The six combinations of treatments are (i) control: 0 mM (SA) + 0 mM NaCl; (ii) 100 mM NaCl; (iii) 200 mM NaCl; (iv) 0.5 mM (SA) + 0 mM NaCl; (v) 0.5 mM (SA) + 100 mM NaCl; and (vi) 0.5 mM (SA) + 200 mM NaCl.

After treatment, the young plants were carefully harvested and then preserved in liquid nitrogen for further investigation. The plant regarded as control (0 mM NaCl) was placed in the growth chamber under a non-salt-stressed condition. A Soil Moisture Meter was used in the measurement of soil moisture level that was conducted on a regular basis (ICT International Pvt Ltd., Australia).

### Dry biomass

After single, combined SA and salt treatment, the total plants were harvested. A centimeter scale was used to measure plant height and leaf length, root and shoot parts were partitioned, and then singly weighed to determine the plant biomass. Root and shoot parts were then dried by placing them in a digital oven for 48 h at 65°C and then weighed again to determine the plant biomass and leaf area ratio^29^.

### Leaf relative water content (RWC)

After single, combined SA and salt stresses treatment, the measurement of RWC was conducted following the formula of ref.^30^: RWC= (FW – DW)/(TW – DW) × 100%. Leaves were weighed to calculate the fresh weight (FW) of stressed mungbean leaves, and the same biological leaves were immediately kept in 100 ml of double distilled water for 12 h to estimate the turgid weight (TW) of the leaves. The leaves were now further placed in envelopes and then oven dried at 65°C for 48 h to estimate the dry weight (DW).

### Gas exchange parameters and photosynthetic pigments

After the stress treatments, the extraction of photosynthetic pigments was done with 80% acetone. A clear supernatant portion was collected for measuring total chlorophyll content and carotenoid after centrifugation for 3 min at 480 × *g* by using the extinction coefficients according to the procedure of ref.^31^. Net photosynthesis rate (PN), photosynthetic quantum yield,^32^ and Soil Plant Analysis Development (SPAD) chlorophyll meter^33^ were measured by using a portable photosynthesis device (LI-6400, LI-CO) between 11 am and 2 pm. Five individual plants were selected from each treatment for measurement. After measuring for photosynthetic efficiency, the upper canopy leaf was detached and then scanned to assess its surface area by using the image analysis system Delta-T Scan (Cambridge, CB50EJ). The data from the gas exchange parameters were estimated based on the specific efficient photosynthetic area.

### Malondialdehyde (MDA) and proline content

We determined the MDA content with thiobarbituric acid (TBA), following the procedure of ref.^34^. The leaves were homogenized in 1% (w/v) trichloroacetic acid (TCA). After centrifugation, to 1 ml of supernatant, 4 ml of 20% TCA containing 0.5% (w/v) TBA was added. The mixture was incubated at 95°C for 30 min. The absorbance was measured at 532 nm and 600 nm, and the concentration of MDA was calculated by using the extinction coefficient of 155 mM per cm.

The method of ref.^35^ was used to estimate proline content using a spectrophotometer (UV752 N, Shanghai Precision & Scientific Instrument Co.). Approximately 0.5 g of plant material was homogenized in 10 ml of 3% aqueous sulfosalicylic acid and the homogenate filtered through Whatman 2 filter paper. Then, 2 ml of filtrate was mixed with 2 ml acldninhdrin and 2 ml of glacial acetic acid in a test tube for 1 hat 100°C and the reaction terminated in an ice bath. The reaction mixture was extracted with 4 ml toluene, mixed vigorously with a test tube stirrer for 15–20 s. The chromophore containing toluene was aspirated from the aqueous phase, warmed to room temperature, and the absorbance read at 520 nm using toluene for a blank. The proline concentration was determined from a standard curve and calculated on a fresh weight basis as follows: proline (mg. g − 1 FW) = [(μg proline/ml × 4 ml toluene)/(0.5 g sample/2.5)]/1000.

### Estimation of electrolyte leakage, antioxidant enzyme activity, and protein content

Electrolyte leakage (EL) were measured as previously described according to ref.^36^. 0.2 g of leaves were cut into pieces of approximately 2 cm^2^ and incubated with 5 ml distilled water in sealed tubes for 4 hat room temperature. The conductivity of the bathing solution was measured with a conductivity meter after which the sealed tubes with the leaves were incubated in a water bath at 95°C for 25 min and cooled down to room temperature. The final conductivity of the bathing solution was measured and for each measurement electrolyte leakage was expressed as percentage leakage according to the following formula:

$$\;E.L(\%)=(\;\frac{Initial\;conductivity}{Final\;conductivity\;})\times 100$$

The methods of ref.^37^ were used to measure POD. The enzyme extract (0.5 ml) was mixed with 1 ml phosphate buffer (0.1 M), 1 ml pyrogallol (0.01 M), and 1 ml H_2_O_2_ (0.05 M) and then incubated (5 min at 25°C). To stop reaction, H_2_SO_4_ (2.5 N) was added and absorbance of the samples were immediately calculated at 420 nm. Catalase (CAT) activity was measured by the method of ref.^38^. The total mixture (3.0 ml) comprised of enzyme extract (100 μl), H_2_O_2_ (100 μl; 300 mM) and 50 mM phosphate buffer (2.8 ml) with 2 mM EDTA (pH 7.0) used for the determination of the CAT activity by taking the absorbance readings at 240 nm using the reduction of H_2_O_2_. The protein content was determined according to the method of ref.^39^. A standard curve was plotted using bovine serum albumin as the standard. Then, 1 g of tissue was extracted in 0.1 M phosphate buffer (pH 7.0) and centrifuged at 15,000 × *g* at 4°C. The supernatant obtained was used for analysis and absorbance was read at 660 nm using water as blank.

### Statistical analysis

Five individual plants from each treatment were taken to investigate the interaction of SA and salt treatment on evaluated biochemical and morphophysiological parameters. The data represented here are means ± SE, and two-way ANOVA was used and divided using Tukey’s post hoc test at *p* > 0.05 level. The linear correlation analysis was carried out between tested parameters in this study with the use of SPSS statistical software developed by IBM, USA, Ver. 18, and graphs were plotted with Sigma Plot (16.0) and R software created by the R core team, Austria.

## Results

### Preliminary evaluation for salinity stress tolerance in mungbean varieties

Our varieties showed differences in genetic variability in the tested traits after 14 d of salt treatment using 50 mM NaCl concentration, and we selected this specific salt concentration for calculating growth stress indices and other analyses. These varieties showed clear differences for three stress tolerance indices (STI, ST, SWP) (Figure 1). It was evident that Var. 145, Var. 155, and Var. 144 showed the highest ST values, displaying that these varieties revealed a smaller reduction in the relative growth rate and exhibited the highest tolerance capacity for salinity stress condition; while Var. 158, Var. 159, Var. 146, and Var. 160 revealed the lowest ST (Figure 1a) value, showing that these varieties revealed the highest salt susceptibility. Likewise the same results were recorded for STI (Figure 1b) and SWP (Figure 1c). Overall, Var. 160, Var. 159, Var. 154, Var. 153, and Var. 160 exhibited the highest STI (Figure 1b) and SWP (Figure 1c) values and are regarded as the salt-susceptible varieties compared to the salt-tolerant varieties Var. 145, Var. 155, Var. 144, and Var. 142.

### Plant growth indices

There was a significant effect on SA and salinity stress on plant growth compared to their control plant (Table 1). It was observed that plant height, leaf length, and plant biomass were significantly reduced to 52.2% and 53.3% under 100 mM and 200 mM NaCl treatment compared to its control of 55.5% and 58%, and 56% and 55% increase was observed under the combination of SA, SA + 100 mM, and SA + 200 mM in Var. 145 (Table 1), while the leaf area ratio also showed a similar reduction in Var. 145 by 58.9% and 64.2% at 100 and 200 mM NaCl treatment in contrast to 47%, 44% ,and 41% at SA, SA + 100 mM, and SA +200 mM, respectively (Table 1). Similarly in Var. 155, the plant height, leaf length, and plant biomass showed a significant decrease of 58.6%, 47.3%, and 45.7% under control, 100 mM, and 200 mM NaCl treatment and 59.2%, 57%, and 54% in SA, SA + 100 mM, and SA + 200 mM (Table 1). However, leaf area ratio recorded a significant decrease by 59.2%, 48.3%, and 43.4% under control, 100 mM, and 200 mM NaCl treatment and a slight increase by 61.5%, 58.1%, and 55.2%, respectively, under SA, SA + 100 mM, and SA + 200 mM (Table 1). Overall, the salt stress showed a drastic reduction in biomass under 200 mM NaCl treatment while the combination of SA and salinity slightly increased the biomass.

**Table 1.** Changes in plant height, leaf length, leaf area ratio, and plant biomass on Var. 145 and Var. 155 seedlings after exposure to C, control, SA, salicylic acid, 100 mM NaCl, SA + 100 mM NaCl, 200 mM NaCl, and SA +100 mM NaCl.

	Plant height (cm)	Leaf length (cm)	Leaf area ratio (cm/g)	Plant biomass (g)	
Treatment	Var. 145 Var. 155	Var. 145 Var. 155	Var.145 Var. 155	Var. 145 Var. 155	
C	7.5 ± 0.04b 6.5 ± 1.04a	5.1 ± 0.04a 4.8 ± 0.36b	42.4 ± 3.11b 30.4 ± 7.23a	0.17 ± 0.01a 0.15 ± 0.02a	
SA	8.4 ± 0.06c 7.1 ± 1.45c	5.2 ± 0.06a 5.1 ± 1.06c	44.8 ± 2.99b 32.8 ± 8.14b	0.19 ± 0.02b 0.18 ± 0.02b	
100 mM	7.3 ± 0.05b 6.2 ± 1.13b	3.5 ± 0.02b 3.8 ± 0.75a	32.7 ± 5.21a 29.6 ± 7.12a	0.11 ± 0.03a 0.13 ± 0.01a	
SA +100 mM	6.2 ± 0.07b 5.7 ± 0.76b	4.1 ± 0.02c 4.3 ± 1.27b	39.3 ± 7.12b 30.6 ± 9.35a	0.12 ± 0.02a 0.14 ± 0.02a	
200 mM	5.8 ± 0.02a 4.9 ± 0.79a	3.2 ± 0.05a 3.7 ± 1.99a	26.9 ± 5.89a 17.6 ± 5.13c	0.09 ± 0.01b 0.08 ± 0.01c	
SA +200 mM	4.3 ± 0.03a 5.7 ± 0.84b	3.5 ± 0.06b 3.9 ± 1.07a	28.7 ± 9.34b 18.2 ± 3.26b	0.08 ± 0.02a 0.15 ± 0.02b	

Values presented are mean ± SE (*n* = 5); different alphabets denote significant mean difference using ANOVA (*P* ≤ 0.05) by Tukey’s test.

### Photosynthetic pigments

This work was conducted on two munbgean varieties under the interaction of SA and salt stress, and the observed results were recorded after 21 dto investigate the effect of salt stress on the threshold that the seedlings could not withstand survival. The experiments demonstrate how two varieties of munbgean were subjected to two levels of NaCl-supplemented concentrations, thereby revealing significant differences in responses to the NaCl treatment. The lethal impacts of salt were noticed at 100 mM, 200 mM NaCl and in combination with SA +100 mM and SA +200 mM after 21 dof salt treatment. Salt stress affected Chl content in mungbean leaves. The amount of Chl level significantly reduced as salt concentration increased (Table 2). At 200 mM in Var. 145, however, both NaCl treatments caused a significant reduction of 43% under 200 mM salt treatment in Chl *a* content, while in Var. 155 a slight reduction of 32% was recorded in the mungbean seedlings after treatment with 100 mM NaCl treatment (Table 2). Overall, increase in salt stress level with SA treatment revealed a significant decrease in Chl *b* compared to its control. After 200 mM NaCl treatment, we observed that a decrease of 29% was observed in Var. 155 compared to a significant decrease of 47% in Var. 145 (Table 2). Significant low values were recorded after 200 mM treatment and decrease from SA + 100 mM and SA + 200 mM. Chl *a* content showed a slight change with decrease of 11% and 34% in Var. 155 and Var. 145, respectively.

**Table 2.** Changes in chlorophyll *a* and *b* on Var. 145 and Var. 155 seedlings after exposure to C, control, SA, salicylic acid, 100 mM NaCl, SA +100 mM NaCl, 200 mM NaCl, and SA +100 mM NaCl.

	Var. 145		Var. 155	
Treatment	Chlorophyll *a*	Chlorophyll *b*	Chlorophyll *a*	Chlorophyll *b*
C	0.71 ± 0.06a	1.9 ± 0.31b	0.27 ± 0.06a	2.21 ± 0.94c
SA	1.56 ± 0.31b	3.04 ± 0.97c	1.7 ± 0.84b	3.8 ± 1.06d
100 mM	0.21 ± 0.04a	1.53 ± 0.76a	0.45 ± 0.09a	1.85 ± 0.71a
SA + 100 mM	0.74 ± 0.09b	2.78 ± 0.79b	1.77 ± 0.62b	2.85 ± 1.05b
200 mM	0.33 ± 0.05a	1.8 ± 0.76a	0.62 ± 0.13a	1.57 ± 0.83a
SA + 200 mM	0.91 ± 0.11b	4.04 ± 1.07d	2.3 ± 0.96c	2.83 ± 0.95c

Values presented are mean ± SE (*n* = 5); different alphabets denote significant mean difference using ANOVA (*P* ≤ 0.05) by Tukey’s test.

### Relative water content (RWC %) and gas exchange parameters

**Figure 2.**
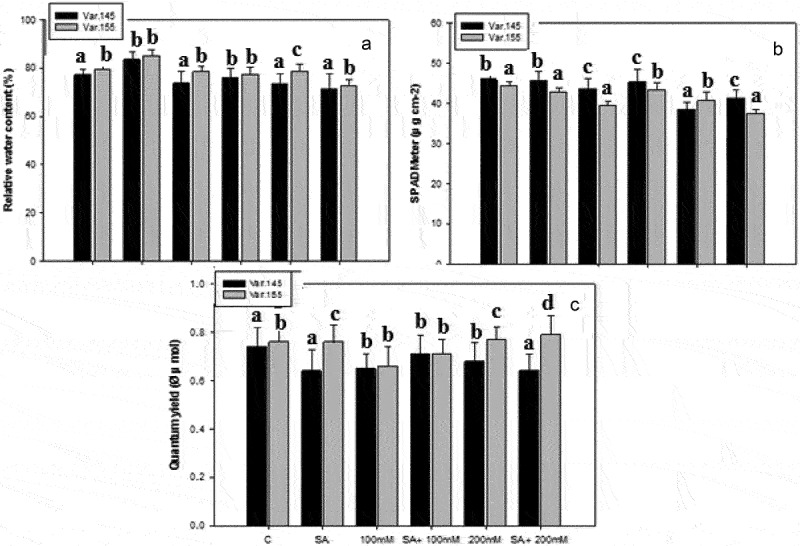
Changes in (A) RWC, (B) SPAD meter, and (C) photosynthetic quantum yield on Var. 145 and Var. 155 seedlings after exposed to C, control, SA, salicylic acid, 100 mM NaCl, SA +100 mM NaCl, 200 mM NaCl, and SA +100 mM NaCl. Values presented are mean ± SE (*n* = 5); different alphabets denote significant mean difference using ANOVA (*P* ≤ 0.05) by Tukey’s test.

The effect of single or combine SA and salinity stress showed a significant impact on RWC content (Figure 2). Control plants induced an increase in RWC but the highest increase was seen in SA-treated plants (Figure 2a). Moreover, RWC showed a gradual decrease under progressive salt stress conditions and the highest reduction was measured under 200 mM NaCl treatment (Figure 2a). The measurement of gas exchange parameters as recorded by dual-PAM in 21-d-old plants under the synergistic interaction of SA and salt stress, viz., control, 100 mM, and 200 mM NaCl treatments (Figure 2). The SPAD showed a higher value in Var. 155 than Var. 145 (Figure 2). Subsequently, the SPAD values showed a reduction of 83% and 72% in Var. 145 (Figure 2b) and Var. 155 (Figure 2b) respectively under SA and salt stress treatments. A drastic reduction in Var. 145 was recorded with the use of cardinal point under NaCl stress in munbgean seedlings (Figure 2b). Likewise, the activity of photosynthetic quantum yield of PS Il recorded a low value (Figure 2c). The deleterious impact of NaCl showed a gradual increase after 3 d with 200 mM NaCl concentration with SA treatment applied, i.e SA + 100 mM and SA + 200 mM.

### Oxidative stress parameters

**Figure 3.**
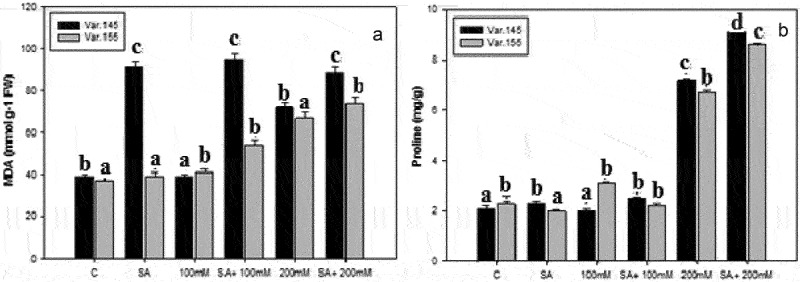
Changes in accumulation of (a) MDA and (b) proline on Var. 145 and Var. 155 seedlings after exposed to C, control, SA, salicylic acid, 100 mM NaCl, SA + 100 mM NaCl, 200 mM NaCl, and SA +100 mM NaCl. Values presented are mean ± SE (*n* = 5); different alphabets denote significant mean difference using ANOVA (*P* ≤ 0.05) by Tukey’s test.

The level of malondialdehyde (MDA) content in the leaves of two mungbean varieties showed a remarkable variation in the tested mungbean varieties (Figure 3a). A clear significant impact of NaCl on the level of MDA is described in Figure 3 in contrast to their control plants. We observed that the concentration of MDA reduced under 200 mM NaCl concentrations in Var. 155, showing a decrease according to concentration dependent. The control plants revealed a gradual decrease in the activities of MDA in both varieties. Moreover, Var. 145 recorded a higher activity of MDA (Figure 3a) compared to Var. 155 (Figure 3a) when treated with SA + NaCl, suggesting more sensitivity to cell damage. A decline was also recorded in both varieties at 200 mM NaCl treatment.

Our data here shows the effect of SA and salinity on the proline content. There was a remarkable upregulation in the level of proline activity at all salt concentrations tested (Figure 3). A significant low level of proline was revealed in the control plants of the two varieties of mungbean (Figure 3b). However, an increase in proline content was recorded in Var. 155 (Figure 3b) more than in Var. 145 (Figure 3b) at SA +100 mM and SA + 200 mM, respectively.

### Antioxidant enzymes activity and compatible soluble protein

**Figure 4.**
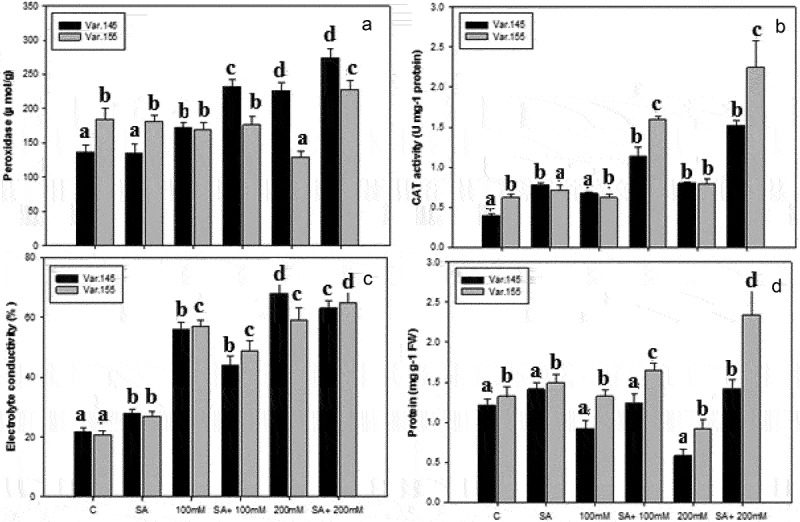
Changes in (a) peroxidase, (b) catalase activity, (c) electrolyte leakage, and (d) protein content on Var. 145 and Var. 155 seedlings after exposed to C, control, SA, salicylic acid, 100 mM NaCl, SA +100 mM NaCl, 200 mM NaCl and SA + 100 mM NaCl. Values presented are mean ± SE (*n* = 5); different alphabets denote significant mean difference using ANOVA (*P* ≤ 0.05) by Tukey’s test.

The activity of POD in Var. 145 revealed different responses with the increase in the concentration of salt in contrast to Var. 155 and their control. In Var. 155, a pronounced increase was observed in the activity of POD more than in their control plants (Figure 4a). A remarkable increase in POD activity was seen in Var. 155 gradually up to the concentration of 100 mM NaCl and SA + 100 mM and SA + 200 mM, while the activity reduced at 200 mM NaCl, but SA + 200 mM recorded a higher activity than its control (Figure 4a). Contrastingly, in Var. 145, combination of SA + NaCl treatment caused an induction in the activity of POD but at 200 mM NaCl treatment showed a reduction and increased at SA + 200 mM (Figure 4b). An increase in CAT activity was seen in Var. 155 (Figure 4b) up to the concentration of 100 mM NaCl and SA + 100 mM and SA + 200 mM, while the activity reduced at 200 mM NaCl, but SA + 200 mM recorded a higher activity than its control as similar in Var. 145 (Figure 4b).

The activity of EL revealed a significant increase in Var. 155 (Figure 4c) more than in Var. 145 (Figure 4c) and likewise increased as salt concentration gradually increased.

The effect of salinity and SA on the soluble protein content in Var. 155 and Var. 145 showed a significant difference in comparison to their control plant in Figure 4. We observed an increase in the level of protein content under the salt stress concentrations for the two varieties. The Var. 155 recorded a significant increase in soluble protein content particularly at SA + 100 mM and SA + 200 mM (Figure 4d) than in Var. 145 (Figure 4d) under salt stress treatments.

### Morphophysiological characteristics and principal component analysis (PCA)

**Figure 5.**
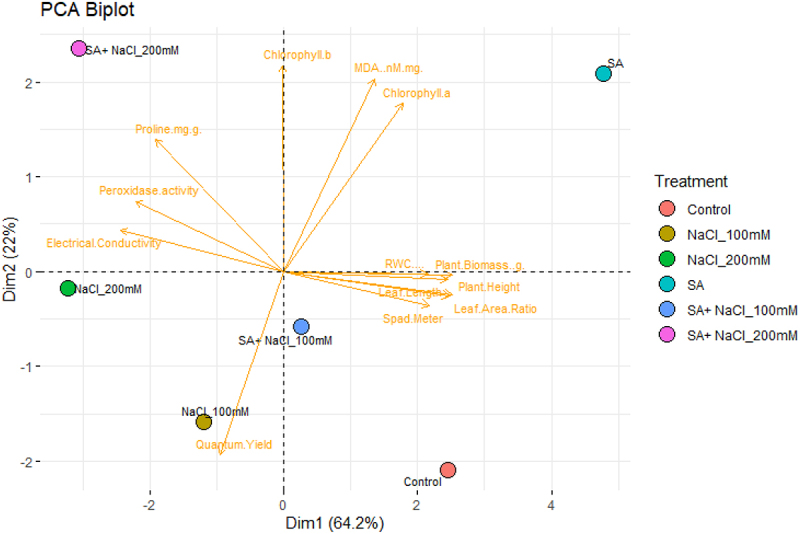
Principal component analysis (PCA) on morphological, biochemical, and physiological parameters of Var. 145 after exposed to C, control, SA, salicylic acid, 100 mM NaCl, SA + 100 mM NaCl, 200 mM NaCl, and SA +100 mM NaCl.

**Figure 6.**
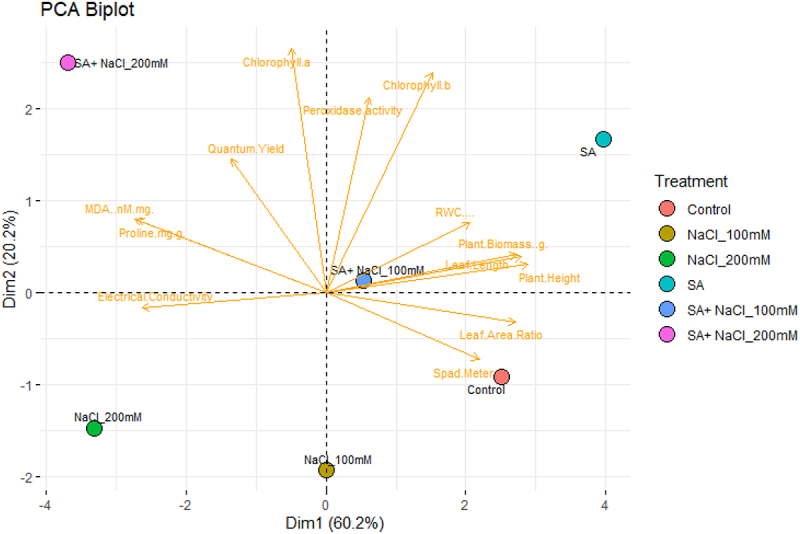
Principal component analysis (PCA) on morphological, biochemical, and physiological parameters of Var. 155 after exposed to C, control, SA, salicylic acid, 100 mM NaCl, SA +100 mM NaCl, 200 mM NaCl and SA +100 mM NaCl.

PCA was used to analyze the significant correlation of the above parameters that were initially subjected to correlation analysis (Figures 5 and 6). PCA shows that the first and second components computed for 86.2% and 80.4% of the total variation in (Figures 5 and 6). The complete variability is 64.2% and 22% (Figure 5) and 60.2% and 20.2% (Figure 6), respectively. The SA and salinity stress conducted in this work indicates that SA and salinity stress revealed a detrimental effect on the development of mungbean. Morphological parameters, gas exchange parameters, photosynthetic pigments, and POD and CAT activities were categorized collectively and then showed a positive correlation between the tested parameters. Also, MDA activities, and protein and oxidative stress parameters were further grouped and revealed a positive correlation with each other.

## Discussion

The resulting effect of salinity stress on the growth of mungbean plants displayed the condition in which the plant develops, the duration of stress applied, and adaptive responses with great effects as seen in salt susceptible variety.

### SA and salinity treatment showed the maximum effect on plant growth

The decline in plant biomass/growth is the most common stress response. Ref. ^40^ reported that most plants reorganize their secondary energy from anabolic processes and biomass accumulation to the stimulation of stress changes. We observed that Var. 145 was more susceptible to salinity more than Var. 155. Our study describes the behavior of two mungbean varieties, Var. 155 and Var. 145, to interaction of saline concentrations and SA. Var. 155 was shown to possess tolerant ability to salinity stress compared to Var. 145. The positive effect of SA application on growth yield as seen in 100 + SA and 200 + SA plants was reported by refs.^41–43^ on the alleviating function of SA on growth yield limited by drought and salinity stresses on varying growth indices. Similarly, ref.^44^ reported that biomass accumulation in the plant shoots of SA treated *Glycine max* seedlings-maintained carbon addition in alleviating water stress. Ref.^45^ reported that pretreatment of rice seedlings with SA at germination stage under salt stress was observed to increase the shoot and root lengths, leading to greater salt resistance. Our result have been able to compare with the report of ref.^44^ on the increase in biomass weight due to the alleviating effect of salicylic acid under salinity stress.

### Gas exchange parameters and photosynthesis pigments content were reduced in mungbean during SA and salinity stress

In this experiment, salinity revealed a decline in photosynthetic performance, quantum yield, and SPAD in the two varieties recorded a higher reduction in Var. 145, which suggests its high sensitivity to salinity stress and therefore causing depletion to the photosynthetic mechanism. However, most plants exposed to saline stress tends to show stress from high light; therefore, the combined effect caused a great damage in photosynthetic performance as shown by the destruction of the proteins in the thylakoid membrane. This suggests that the reduction in plant growth is a result of osmotic stress. Ref. ^46^ reported that reduction in growth caused by osmotic stress usually occurs at early developmental stages when exposed to salinity stress. Ref. ^47^ also reported that two *Triticum aestivum* cultivars showed two category of photoinhibition damage under salinity treatment: the first category shows that the photosynthetic efficiency was reduced gradually, while the second category showed that the photosynthetic energy was fast and followed by a gradual decrease of the energy performance in photosystem II, which is actively associated to detrimental impact of salt stress. Several studies have observed that salt causes plant stomatal closure and decreases intercellular CO_2_ level in the early stages of stress treatment, while in the latter stages, the chlorophyll synthesis is blocked and chloroplasts become destroyed, altering photosynthesis and leading to decrease in biomass, retarded plant growth, and cell death ^48–50^. Ref. ^51^ reported that the impact of salinity stress on the photosystem II complex is majorly because of the repair of the PS II complex through hinderance on the activity of transcription and translation processes. SA has been reported by ref.^18^ to protect most plants against environmental stresses like salinity and drought by maintaining photosynthesis performance. Similarly, ref.^52^ reported on the different SA concentrations on the photosynthetic quantum yield on the growth of *Cucumis sativa*s under saline stress. Ref. ^53^ found that SA can maintain vital plant physiological processes like antioxidant defense system control, nitrogen metabolism, photosynthesis, and improved water use efficiency. Our study has demonstrated the effects of salinity stress on the growth of mungbean plants due to the photoinhibition of PS II induced by stimulated NaCl. This is because salinity stress could have directly acted on sites that are important for the preservation of PS II systems against photoinhibition process. The elevated NaCl content led to the depletion on Chl content, and this might be due to the inhibition of chlorophyll biosynthesis, thereby increasing degradation activities under salinity stress^54^. The increase in salt stress treatment is closely linked to the catabolic breakdown of Chl, which is a visible symptom of salinity stress^55^. Our study revealed that there is a decrease in Chl content of both mungbean varieties as NaCl concentration increased in a dependent manner. It was observed that the Chl a in *Panicum miliaceum* was reduced due to salt applications in landraces that have distinct salt tolerance characteristics^56^. Similarly, an increase in Chl content was seen in *Cucumis* sp., broad bean, rice, and wheat plants under saline stress treatment^57,58^. Ref. ^59^ reported on the exogenous application of SA on the growth, photosynthetic efficiency, and oxidative repair in *Vigna radiata* seedlings under salt stress condition. SA has been found to enhance photosynthetic performance and the antioxidant defense activity to increase yield attributes in the ASD16 and BR26 *Oryza sativa* lines^60, 61^. Overall, the impact of salt application caused depletion of Chl pigment in Var. 145 than in Var. 155.

### Response to osmotic adaptation in mungbean subjected to SA and salinity stress

The salt tolerance characteristics in Var. 155 were related to the antioxidant impact of osmolytes and not their involvement to osmotic adaptation. Ref. ^62^ reported on the strong relationship between increase in proline levels and the ability to withstand the effects of salinity. Therefore, proline is the main source of nitrogen and energy after stress metabolism and the proline accumulated has the ability to supply cellular energy for plant growth and conferring salt tolerance. Also, the osmolytes play a role in preserving macromolecules by maintaining the protein formed and combating reactive oxygen species generated under salt stress conditions^63^. Our results show that the two mungbean varieties elevated the levels of proline as concentration of salt stress increased, Var. 155 recorded higher levels of proline content, thereby indicating an effective function of these amino acids as osmoprotectant when exposed to salinity stress condition. SA increased toward proline accumulation to maintain redox buffering and osmoregulation in mungbean development^43,64^. Ref. ^65^ found that the concentration of proline content has been used as vital physiological indices to quantify different stress resistance in plants. Under exogenously applied SA treatment, the proline metabolism is significantly disrupted, resulting in the maintenance of turgor by the uptake of higher levels of free proline in lentils to increase their salt tolerance capacity^56^. Our result showed that Var. 155 revealed a higher resilience capacity under salt stress treatment as assessed by photosynthetic performance, accumulation of osmoprotectants, and antioxidant defense metabolism induced by SA compared to Var. 145. Protein synthesis is an important mechanism that is affected by salinity stress in plants. Ref. ^66^ describes protein as a vital indicator of the health status of plants. We observed an increase in the soluble protein content, which suggests tolerance responses such as increase in proline and antioxidative enzymes activity, thereby enhancing salt tolerance ability by SA treatment. Thus, protein synthesis is increased as the stress treatment increases. Similarly, decrease in the growth of mungbean plant could suggest low rates of protein synthesis, which is mostly related by the biosynthesis of proteins that are induced under stress. Moreover, ref. ^61^ reported that when protein levels reduced within the cells, it, therefore, recovers the physiological mechanisms in plants after SA treatment^67^. The gradual decrease in protein under a high salt level causes a reduction in photosynthesis performance and could be a characteristic of salinity stress in plants. Refs. ^68,69^ found that a high level of soluble proteins was recorded in salt-resistant genotypes of finger millet, sunflower, rice, and barley. Likewise, it was observed that SA treatment induced the mungbean seedlings to stabilize the homeostasis by protein synthesis^36,70^. The production of ROS is the most expected response that occurs during stress conditions.

### Increased antioxidant enzyme activities to mungbean in response to SA and salinity stresses

Quick response of reactive oxygen species (ROS) effect results in deleterious cellular changes during oxidative stress situation, with MDA and cellular depletion to DNA and proteins and probably lead to plant cell death^71^. MDA is an indicator for determination of damage to organelle membranes and plasmalemma under increased environmental stress conditions. The results reported here showed that the MDA accumulated increased in Var. 145 compared to Var. 155 under SA + salt stress treatment, suggesting a significant high level of MDA activity in Var. 145. The higher activities of POD, EL, and CAT and lowest degree of cell membrane damage revealed in salt-treated plants of Var. 155 showed that this variety possesses a potent capacity for combating ROS produced by salinity treatment compared to Var. 145.

The activities of antioxidant enzymes are closely linked to lipid peroxidation, e.g. the induction of CAT, EL, and POD induces adaptation to several stress tolerance and decrease in the activity of MDA. The combating of ROS is linked with two vital activities of antioxidant enzymes responsible for defense processes in the cell (especially in catalase, superoxide dismutase, and glutathione peroxidase) and the availability of other antioxidative substances, such as proline and mannitol^72^. Ref. ^73^ reported on the role of antioxidative enzymes in *Lycopersicon esculentus* and on wild type salt-resistant *Lycopersicon pennellii* under salt stress treatment. The activities of POD and CAT in *L. pennellii* were higher compared to *L. esculentum* after exposure to 100 mM NaCl treatment. In the present study, activities of enzymes revealed a gradual increase in the two mungbean varieties at the imposed tested salt concentrations. Ref. ^74^ reported that some enzymes are involved in oxidative stress, like catalase (CAT), superoxide dismutase (SOD), glutathione reductase (GR), and peroxidase (POX), that increase their enzymatic activities under saline stress treatment. In line with the physiological process, exogenously SA-treated plants showed increased expression level in the transcripts of several antioxidant constituents, like glutathione peroxidase (GPX1, 2), dehydroascorbate reductase (DHAR), glutathione S-transferase (GST1, 2), and glutathione synthetase (GS) under saline stress^75^.In the same vein, ref. ^42^ reported on the impacts of exogenous SA on photochemical activity, phenolic metabolism, and antioxidative responses of Fragaria under salinity stress. Several researchers like refs.^76,77^ documented the effect of salicylic acid on antioxidant defense and ROS regulation in barley and *Egletes viscose*. Ref. ^78^ found that various researchers have documented the activities of APX, CAT, and SOD exposed to salinity stress in the two varieties. These results could highly suggest the possibility that the activities of the above antioxidant enzymes are useful in mungbean to reduce cell membrane damage caused by salinity stress, thereby preserving the cell membrane from impairment. The progressive increase in the activities of the antioxidant enzymes under salinity stress is closely linked to the improved tolerance capacity to salinity stress condition^79^. A rapid increase in activity of POD could reveal that POD is said to be an important enzyme detoxifying hydrogen peroxide in Var. 155 after exposure to salt stress condition. The increase in CAT activity showed a twofold elevation compared in POD may contribute in stabilizing a series of cellular hydrogen peroxides. Herein, our study suggests positive inclusion of catalase and peroxidases in H_2_O_2_ combating antioxidant enzymes in conferring salt tolerance capacity in mungbean seedlings. This shows that the activities of EL and CAT in Var. 155 revealed a significant increase under salt stress. The energy recovered through enhanced photosynthetic performance under SA application was useful in protein, carbon, and nitrogen metabolism producing defense action against salinity. POD and CAT level revealed gradual increase in salt-stressed seedlings under SA treatment, thereby providing a potent ROS combating system under salt stress in mungbean development. Similarly, ref. ^80^ found that SA treatment induced antioxidant defense potency of plants by enhancing the enzyme activities and antioxidant series.

### Analyzing the tolerant ability of mungbean to SA and salinity stresses

The report by ref. ^81^ states that the PCA assessment gives a general clarification of the interconnection between all parameters evaluated, and we detected what index trait can be considered as a single, compatible, or added method for evaluating the interactive effect of SA on mungbean growth under salinity stress conditions, single or combined. Our results showed that morphological growth indices (plant height, leaf length, leaf area ratio, and biomass), photosynthetic pigments content (Chl *a* and *b* content), gas exchange parameters (SPAD and quantum yield), and APX activity were categorized jointly. Overall, CAT was specifically grouped with MDA and POD content and protein. Hence, the parameters represented in each category are positively correlated and are selected as an integral screening tool for evaluating the interaction of mungbean to SA and salinity stress conditions, single or combined.

Also, the negative correlation existing between these two categories indicates that salinity and SA, single or combined, could cause cell membrane damage through the gradual reduction of extreme accumulation of MDA and POD, thereby affecting photosynthesis performance and growth of mungbean. Therefore, mungbean can adapt to salt stress because of the SA treatment and induction of CAT activity. Gas exchange parameters, photosynthetic pigments content, soluble protein, CAT activity, MDA, and POD can be used as basis on the alleviating effect of SA on the growth of mungbean under salinity stress condition.

## Conclusion

SA induced modulations to help most plants achieve developmental equilibrium and energy to adjust with stress conditions. SA modulates the metabolic and physiological processes toward the acclimation and survival of mungbean seedlings exposed to salt stress condition. Our results here showed that Var. 155 performed better than Var. 145 under SA and salinity interaction, as observed by low accumulation of proline, photoinhibition, and high antioxidant enzyme activities. Furthermore, due to the pronounced increase in antioxidant enzyme activities, chlorophyll content and the process of adjustment to stabilize maximum efficiency of photosystem II was observed in Var. 155 under the different salinity levels. The processes underlying growth of mungbean seedlings under interaction of salt and SA hasten the plants by improving the stress tolerance capacity to acquire yield. In this work, we suggest SPAD chlorophyll meter as a valuable tool to evaluate crop lines and could be used in physiological responses to determine the photosynthetic performance on plants under salt stress treatment. Therefore, our findings indicate that multiple usage of SPAD chlorophyll meter could be employed as a vital tool to evaluate the responses of most plants to abiotic stressors and in identifying stress-tolerant plant species. If we can analyze how plants refuse the increase of SA by each stress and identify the promoters specifically induced by salt stress in the SA synthesis pathway, this will give us a better understanding of plant responses to salt stress and applied SA treatment more effectively. Further research should emphasize the ameliorating function of SA from salt stress condition with the main objective of enhancing yield performance in any stress-prone environment.
